# Cervical Myelopathy Associated With Deep Neck Muscle Rhabdomyolysis After Polysubstance Abuse: A Case Report

**DOI:** 10.7759/cureus.108566

**Published:** 2026-05-09

**Authors:** Sofia C Pfannenbecker, Mohan Prabu, Olivia Wei, Ramin Javan

**Affiliations:** 1 Neuroscience, George Washington University, Washington, USA; 2 Neurology and Epilepsy, Ascension St. Vincent Kokomo, Kokomo, USA; 3 Radiology, George Washington University, Washington, USA

**Keywords:** cervical myelopathy, compartment syndrome, deep neck muscle rhabdomyolysis, polysubstance abuse, rhabdomyolysis, spinal cord infarction

## Abstract

Rhabdomyolysis is a potentially life-threatening syndrome caused by skeletal muscle breakdown and the release of intracellular contents into circulation, often resulting in complications such as acute kidney injury. Although it most commonly affects the limbs, rhabdomyolysis of the deep neck muscles is rare and can lead to secondary cervical myelopathy. Recent reports have highlighted a strong association with polysubstance abuse, particularly combinations of opioids with sedatives such as benzodiazepines or pregabalin.

We describe a 41-year-old female patient who developed acute quadriparesis following polysubstance use involving pregabalin, methamphetamine, buprenorphine, and alcohol. MRI revealed a longitudinal T2-hyperintense cervical cord lesion from C2 to T3 with marked paravertebral muscle edema, consistent with deep neck muscle rhabdomyolysis. Laboratory findings showed a creatine phosphokinase peak of 16,093 U/L, which normalized with corticosteroid therapy and intravenous hydration. Despite normalization of laboratory values, the patient had persistent limb weakness at discharge due to venous hypertensive myelopathy, inadvertently caused by deep cervical rhabdomyolysis. This case reinforces emerging evidence that deep cervical muscle rhabdomyolysis can lead to venous hypertensive myelopathy through a compartment-syndrome-like mechanism. Awareness of this presentation is essential, as early MRI of both the spinal cord and paravertebral musculature may enable timely diagnosis and improved outcomes in polysubstance-related myelopathy.

## Introduction

Rhabdomyolysis is a clinical syndrome characterized by the breakdown of skeletal muscle, resulting in the release of intracellular components, including creatine kinase, potassium, and myoglobin, into the systemic circulation [1]. It is a well-recognized condition encountered worldwide in both emergency and inpatient settings. Although overall incidence varies by population and etiology, with rising recognition over the past two decades, large U.S. database studies estimate approximately 0.7 cases per 100,000 persons annually presenting to emergency departments [2]. The condition has a broad range of causes, including trauma, exertion, infections, metabolic disorders, toxins, and drugs [3]. Extremity muscle involvement is most typical; however, involvement of the cervical or deep neck musculature is rare and has primarily been described in case reports and small case series. Rhabdomyolysis of the deep neck musculature has only recently been recognized as a distinct entity that may lead to secondary cervical myelopathy, and its true incidence is likely underrecognized [4].

Substance-related rhabdomyolysis is well documented. Methamphetamine abuse frequently leads to severe rhabdomyolysis and kidney injury [1]. Opioid use and withdrawal have also been implicated [5]. More recently, pregabalin has emerged as an independent contributor, particularly when combined with opioids or statins [6,7]. Designer benzodiazepines have likewise been reported as precipitants of severe rhabdomyolysis [8]. These associations highlight that the range of substances capable of triggering muscle breakdown continues to expand.

The clinical importance of recognizing rhabdomyolysis in the neck is underscored by its potential to cause spinal cord compression or vascular injury. Honkaniemi et al. described eight young men who developed cervical myelopathy with adjacent paravertebral rhabdomyolysis after polysubstance use (e.g., opioids, stimulants, and alcohol) [4]. Magnetic resonance imaging (MRI) findings consistently demonstrated hyperintense lesions within the cervical cord, accompanied by abnormalities in the multifidus and semispinalis cervicis and capitis muscles. Additional case reports have confirmed that nontraumatic rhabdomyolysis of head and neck muscles may occur in association with substance use, dehydration, nutritional supplement use, metabolic stress, and other nontraumatic conditions, and can mimic other inflammatory, ischemic, demyelinating, or compressive myelopathies, making imaging a critical diagnostic tool [9].

Pathophysiologically, this syndrome differs from classic flexion myelopathy. Although alcohol-induced cases of compressive myelopathy with prolonged neck flexion have been reported [10], most polysubstance-associated cases occur without extreme cervical positioning. Instead, the prevailing mechanism involves compartment syndrome of the deep paravertebral muscles, which impairs venous outflow and leads to venous hypertensive myelopathy [11]. The resulting spinal cord changes are distinct on MRI, particularly on MRI T2-weighted sequences, where cord hyperintensity and edema may be observed.

Awareness of this presentation is critical, as delayed recognition often results in severe disability or death during follow-up [4]. With polysubstance abuse on the rise, clinicians must consider cervical rhabdomyolysis in patients presenting with acute myelopathy following intoxication and immobilization. This report describes a case of polysubstance-associated deep neck muscle rhabdomyolysis that led to cervical myelopathy and reviews the clinical and radiologic features that aid diagnosis.

## Case presentation

A 41-year-old female patient with a history of polysubstance use (nicotine, buprenorphine/naloxone, methamphetamine, and amphetamine/dextroamphetamine) and alcohol use disorder, bipolar disorder, attention-deficit/hyperactivity disorder, iron-deficiency anemia, and prior thoracic epidural abscess was found unresponsive in her bathroom after ingesting six pregabalin (Lyrica) tablets (the tablet strength was not reported by the patient and was not documented in the medical record) with approximately seven to eight shots of vodka. She additionally reported intranasal use of buprenorphine/naloxone (Suboxone) two days prior to presentation. On arrival, she was febrile (101 °F) and reported profound quadriparesis and neck pain. A formal Poisoning Severity Score was not documented in the medical record. Neurological examination demonstrated near-complete distal paralysis of all four extremities with preserved cranial nerve function and intact sensation above the neck. Deep tendon reflexes were diminished, and she was unable to lift her arms against gravity.

Urine toxicology screening was positive for amphetamine, methamphetamine, and buprenorphine and negative for methadone, cannabis, opiates, oxycodone, phencyclidine (PCP), benzodiazepines, and tricyclic antidepressants. Initial laboratory evaluation, performed in the hospital’s central clinical laboratory using standard automated analyzers, revealed a markedly elevated creatine phosphokinase (CPK) level of 16,093 U/L with aspartate aminotransferase of 349 U/L and alanine aminotransferase of 158 U/L, consistent with severe rhabdomyolysis. Serum electrolytes demonstrated mild hyponatremia (Na 129 mmol/L), which normalized during hospitalization. Cerebrospinal fluid (CSF) analysis demonstrated normal glucose (69 mg/dL) and protein (50 mg/dL), three white blood cells (WBCs), and negative oligoclonal bands. All laboratory values are summarized in Table 1.

**Table 1:** Summary of initial and recovery laboratory findings following polysubstance-related cervical rhabdomyolysis

Laboratory test	Result on admission	Result at day 8 (recovery)	Units	Reference range
Creatine phosphokinase (CPK)	16,093	74	U/L	26–308
Aspartate aminotransferase (AST)	349	—	U/L	10–40
Alanine aminotransferase (ALT)	158	—	U/L	7–56
Sodium (Na)	129 → normalized	—	mmol/L	135–145
Cerebrospinal fluid (CSF) glucose	69	—	mg/dL	40–75
CSF protein	50	—	mg/dL	15–45
White blood cells (WBC), CSF	3	—	cells/µL	0–5
Oligoclonal bands	Negative	—	—	Negative

Further diagnostic evaluation included electroencephalography (EEG), which demonstrated no seizure activity. Transthoracic echocardiography showed normal biventricular function without valvular abnormalities. Computed tomography (CT) of the head and CT imaging of the cervical, thoracic, and lumbar spine revealed no acute abnormalities. Chest X-ray demonstrated no acute cardiopulmonary abnormality. MRI of the brain with and without contrast demonstrated no acute intracranial abnormality.

**Figure 1:**
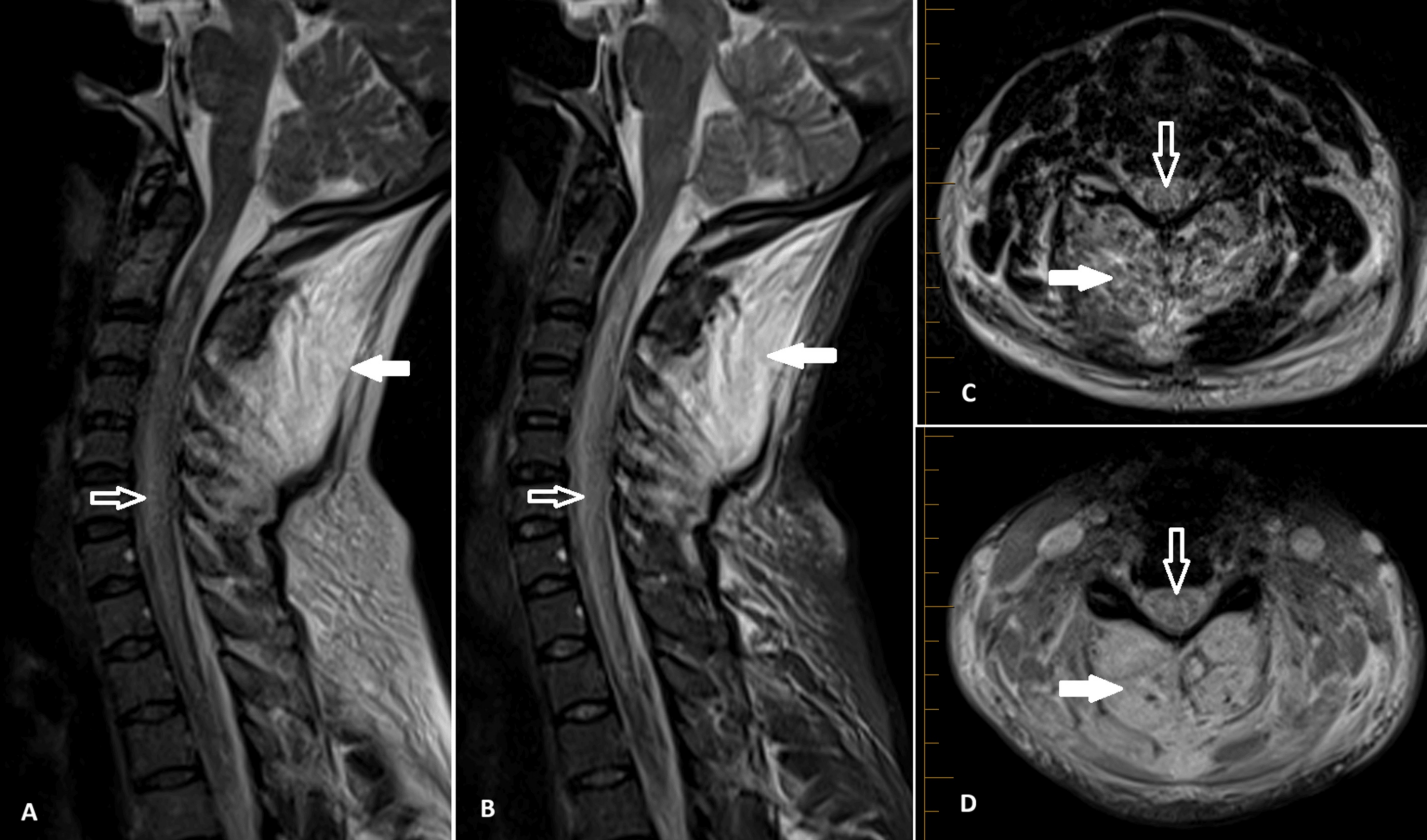
Multi-sequence cervical spine MRI demonstrating extensive paraspinal muscle edema and longitudinal cord edema (C2–T3) Multi-sequence cervical spine MRI, performed using standard clinical imaging protocols, demonstrating extensive paraspinal muscle edema and longitudinal cord edema (C2–T3). Scanner field strength (1.5T vs 3T) was not available in the medical record. A: sagittal T2; B: sagittal Short Tau Inversion Recovery (STIR); C: axial T2; D: axial Gradient Recalled Echo (GRE) images showing marked T2 and STIR signal hyperintensity in the posterior paraspinous musculature and soft tissues compatible with extensive edema (solid/white arrow), most pronounced in the multifidus muscles, suggestive of rhabdomyolysis. There is also marked T2 and STIR signal hyperintensity extending from C2 to the T3 level, indicating extensive cord edema (hollow/black arrow) with cord expansion.

MRI of the cervical spine without contrast demonstrated a longitudinal T2-hyperintense lesion extending from C2 to T3 with mild cord expansion and extensive posterior paraspinal muscle edema involving the multifidus and semispinalis muscles, findings consistent with deep neck muscle rhabdomyolysis (Figure 1).

Infectious and autoimmune evaluation was negative, including human immunodeficiency virus (HIV) testing, hepatitis serologies, and antinuclear antibody (ANA) testing.

The constellation of severe rhabdomyolysis, paravertebral muscle edema, and longitudinal cervical cord hyperintensity supported the diagnosis of cervical myelopathy secondary to deep neck muscle rhabdomyolysis following polysubstance use. Over 10 days, she was treated with high-dose corticosteroids, aggressive intravenous hydration, and supportive care. CPK normalized by day eight (74 U/L), correlating with systemic recovery. Despite biochemical resolution, right-predominant upper-extremity weakness persisted at discharge. At five-month follow-up, the patient demonstrated gradual neurological improvement with assisted ambulation.

## Discussion

This case adds to the growing body of evidence that deep neck muscle rhabdomyolysis can lead to cervical myelopathy, a condition that until recently was rarely recognized in clinical practice. Honkaniemi et al. [4] first highlighted a series of young men who developed profound tetraparesis after polysubstance use, with MRI showing cervical cord lesions and adjacent paravertebral muscle abnormalities. The consistency of these findings across multiple patients suggests that this syndrome is a distinct clinical entity, most likely underdiagnosed due to its overlapping presentation with other forms of acute myelopathy.

In the present case, the combination of pregabalin, methamphetamine, and buprenorphine likely contributed to both direct myotoxicity and immobilization-related muscle ischemia. MRI findings mirrored those in previous cases, demonstrating longitudinal cervical cord hyperintensity and extensive paravertebral muscle edema. The absence of enhancement and negative CSF oligoclonal bands helped rule out demyelinating and infectious causes. The CPK peak of 16,093 U/L fell within the upper range of reported values (181-21,995 U/L; median ≈ 6,470 U/L) from Honkaniemi’s cohort [4], suggesting a similar degree of muscular injury. Despite biochemical resolution, neurological recovery remained incomplete.

The pathophysiology appears to involve compartment syndrome of the deep cervical musculature. Swelling within the paravertebral muscles, particularly the multifidus and semispinalis cervicis and capitis, impairs venous outflow and results in venous hypertensive myelopathy rather than arterial infarction [11]. This mechanism accounts for the characteristic T2 hyperintense cord lesions with surrounding hypointense rims observed on MRI [4]. Histological findings from prior reports support this theory, showing muscle necrosis and fascial thickening consistent with compartment syndrome.

Importantly, this syndrome is distinct from classic flexion myelopathy. While Maramattom [10] described alcohol-related acute ptotic myelopathy following extreme cervical flexion, most polysubstance-associated cases, including this one, occur without such positioning. Instead, prolonged immobilization in the setting of intoxication appears to be sufficient to precipitate a cascade involving rhabdomyolysis, compartment syndrome, venous congestion, and subsequent spinal cord injury.

The clinical associations are also notable. Polysubstance use involving opioids combined with sedatives such as pregabalin or benzodiazepines is a recurrent feature [4]. Pregabalin, in particular, has been associated with rhabdomyolysis, both independently [6] and in conjunction with other agents [7]. Designer benzodiazepines have also emerged as precipitants of severe rhabdomyolysis [8]. On the stimulant side, methamphetamine has a well-documented association with rhabdomyolysis and acute kidney injury [1]. Taken together, these findings suggest that multiple classes of substances can predispose patients to rhabdomyolysis, but the combination of opioids with gamma-aminobutyric acid (GABA) agonists appears to carry a particularly high risk for deep cervical muscle involvement.

Radiologic findings serve as a crucial diagnostic clue. MRI consistently demonstrates hyperintense lesions in the cervical spinal cord alongside increased T2 signal in adjacent paravertebral muscles, a pattern described by Honkaniemi et al. [4], Debelmas et al. [9], and Lugo-Fagundo et al. [12]. These imaging features can help differentiate this syndrome from mimics such as transverse myelitis, demyelinating disease, or spinal cord infarction.

Rhabdomyolysis itself has a broad differential etiology, including trauma, prolonged immobilization, drug and toxin exposure, metabolic derangements, infections, seizures, and exertional injury. In polysubstance-associated cases, multiple mechanisms may coexist, including direct myotoxicity, prolonged immobilization, dehydration, and metabolic stress, which can exacerbate muscle injury and complicate identification of the primary trigger [1,3,13]. Rhabdomyolysis also demonstrates wide variability in clinical severity and etiology, and epidemiologic characterization can be challenging because presentations range from mild biochemical abnormalities to life-threatening systemic disease [14]. 

The differential diagnosis for patients presenting with acute weakness and myelopathic signs is similarly broad and includes transverse myelitis, spinal cord infarction, demyelinating disease, compressive lesions, and toxic or metabolic myelopathies. Overlapping clinical and imaging features may delay diagnosis [14].

Beyond neurologic injury, rhabdomyolysis carries systemic risks such as acute kidney injury, electrolyte disturbances, disseminated intravascular coagulation, and cardiac arrhythmias [4]. The release of intracellular contents such as myoglobin and electrolytes into the circulation contributes to systemic toxicity and can lead to multiorgan complications. Recognition of both neurologic and systemic complications is essential for comprehensive management [1,3].

The prognosis remains guarded. In Honkaniemi et al.’s [4] series, several patients died within a few years of onset, and many survivors were left with significant disability. Supportive management, including hydration to prevent renal failure, remains the cornerstone of treatment, while surgical decompression (fasciotomy or laminectomy) has been attempted in select cases with limited success [13].

Ultimately, this case reinforces that deep neck muscle rhabdomyolysis leading to venous hypertensive myelopathy is an underrecognized but clinically distinct consequence of polysubstance abuse. Prompt MRI evaluation and early recognition are critical to improving outcomes and guiding rehabilitation in this otherwise devastating condition.

## Conclusions

Cervical myelopathy associated with deep paravertebral rhabdomyolysis represents an uncommon but severe complication of polysubstance abuse. Recognition of this entity is important because imaging findings can closely mimic those of other causes of acute myelopathy. In this case, the patient developed acute cervical myelopathy after polysubstance exposure, including pregabalin, buprenorphine, and alcohol, with methamphetamine exposure confirmed on urine toxicology screening. MRI demonstrated longitudinal cervical cord T2 hyperintensity with extensive paravertebral muscle edema. A markedly elevated CPK level supported the diagnosis of severe rhabdomyolysis, while additional testing helped rule out demyelinating, infectious, or inflammatory etiologies.

Unlike classic flexion myelopathy, this syndrome develops without sustained cervical flexion and is instead driven by deep paravertebral compartment syndrome leading to venous hypertensive myelopathy. The marked paravertebral muscle edema and longitudinal T2 hyperintensity observed on MRI in this patient are consistent with the proposed mechanism of venous congestion and cord edema described in prior reports. Although her CPK normalized rapidly with treatment, neurological recovery was incomplete, highlighting the risk for persistent deficits. Early cervical MRI and close clinical suspicion are therefore essential for timely diagnosis and management. Improving outcomes may depend on prompt recognition, supportive therapy to prevent renal and spinal sequelae, and targeted strategies to reduce polysubstance-related immobilization and toxicity.
